# Controllable Phase Transformation and Enhanced Photocatalytic Performance of Nano-TiO_2_ by Using Oxalic Acid

**DOI:** 10.3390/nano12173019

**Published:** 2022-08-31

**Authors:** Jiaqi Chen, Jian Gao, Xiaoyang Liu, Pan Wang, Xue Yu, Feng Zhao, Yan Sun, Wei Feng, Qingyuan Wang

**Affiliations:** 1 School of Mechanical Engineering, Chengdu University, Chengdu 610000, China; 2 Institute for Advanced Materials Deformation and Damage from Multi-Scale, Chengdu University, Chengdu 610106, China

**Keywords:** TiO_2_, photocatalyst, inhibitor, phase transformation

## Abstract

Degradation of organic pollutants, especially organic dyes and antibiotics, by semiconductor photocatalysts is an efficient strategy for wastewater treatment. TiO_2_ nanomaterials are considered to be promising photocatalysts due to their high chemical stability, high efficiency and availability. Anatase TiO_2_ generally has superior photocatalytic activity to the rutile phase. However, the anatase phase can be irreversibly transformed to rutile phase when calcined at an elevated temperature. Methods to improve the stability of anatase are especially important for the TiO_2_ gas sensors working at high temperatures. The addition of strong acids can effectively suppress this transformation process. However, these strong acids are relatively expensive, corrosive and environmentally unfriendly. Herein, oxalic acid (OA) as a natural acid was used to control the hydrolysis process of tetrabutyl titanate (TBOT), leading to controllable crystalline phase transformation and reduced crystalline size of TiO_2_ on the nanoscale. What is more, the photocatalytic degradation performances were enhanced continuously when the molar ratio of OA to TBOT increased. The degradation reaction rate constants of CT650-R25 were about 10 times that of CT650-R0. The mechanism study shows that the enhanced photocatalytic activity can be attributed to the improved dispersibility, increased specific surface area and reduced recombination rates of photo-induced charge carriers and decreased energy bands as the concentration of OA increased. Thus, this work provides a simple, mild and effective method for controlling the crystalline forms of nano-TiO_2_ with enhanced photocatalytic performance towards waste water treatment.

## 1. Introduction

With the continuous growth in population and rapid development of the economy, the problem of environmental pollution has increased. Organic dyes such as methyl orange (MO), methylene blue and rhodamine [1], and antibiotics such as tetracycline (TC) [2,3], have become major pollutants affecting the water environment. Untreated organic dyes are often slowly naturally degraded. Since most organic dyes have carcinogenic and mutagenic effects [4], they will cause various diseases after bioaccumulation in the human body through the food chain [5]. On the other hand, antibiotics are widely used in farming and by humans as drugs for the treatment of infectious diseases. However, after entering the human body or animals, 5–90% of the antibiotics are excreted through urine or feces [6]. Antibiotics can easily enter the environment through direct or indirect ways. Many reports have pointed out that a variety of antibiotics have been detected in natural water environments, including oceans, rivers, lakes and groundwater [7,8,9]. At present, the methods for treating organic pollutants in water are photocatalytic technology [10], physical adsorption [11], biological decomposition [12] and other technologies. Among them, photocatalytic degradation technology is an emerging green technology [13,14]. Photocatalysts can degrade organic pollutants into harmless final products, such as carbon dioxide, water and inorganic ions under light radiation [15]. At present, several types of semiconductor metal oxides and sulfides, such as TiO_2_, Cu_2_O, ZnO, CdS and ZnS, have been discovered [16]. TiO_2_ stands out due to its excellent stability, high efficiency and low cost [17,18,19].

The photocatalytic activity of TiO_2_ is affected by many factors, such as crystalline forms [20], specific facets [21], surface morphology [22], heterogeneous structures [23], types of inhibitors and modifiers. Among them, the effect of crystalline forms on the photocatalytic activity of TiO_2_ is particularly important [24]. It is well known that TiO_2_ exists in three crystalline forms, anatase, rutile and brookite [25]. Brookite is rarely used in the photocatalytic applications due to its instability [26]. It is generally believed that anatase has better photocatalytic properties than the more stable rutile [27]. However, the anatase phase can irreversibly be converted to the rutile phase when calcined at elevated temperatures [28]. It is important to study the conditions affecting the phase transition kinetics, especially for applications such as gas sensors and porous gas separation membranes running at high temperatures [29]. Therefore, the factors that suppress this transformation process have been intensively discussed [30]. Many researchers have used various acids, such as hydrochloric acid [31] and sulfuric acid [32], as inhibitors to suppress the transformation. What is more, the addition of acids can effectively control the crystalline form [33], grain size [34], specific surface area and surface oxygen defects of TiO_2_ [35]. However, these strong acid inhibitors are relatively expensive and corrosive for industrial applications. Therefore, our work explored an economical, environmentally friendly and stable method by employing the natural mild oxalic acid (OA) to maintain the excellent photocatalytic ability of anatase at high temperatures.

**Scheme 1:**
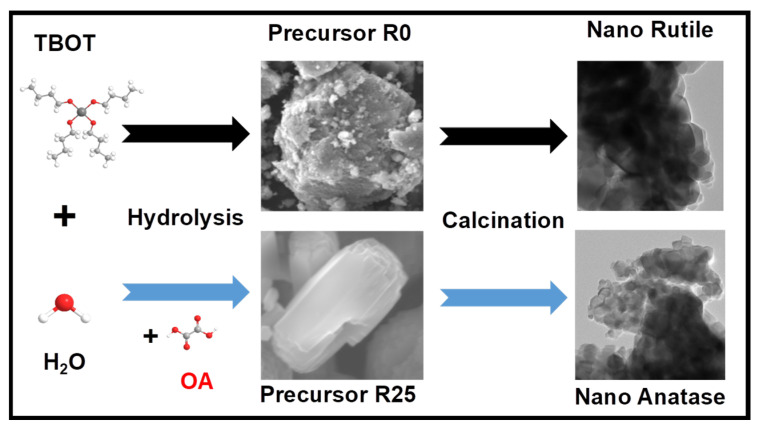
Preparation process of nano-TiO_2_ with the controllable crystalline forms.

In this study, tetrabutyl titanate (TBOT) was used as the titanium source and oxalic acid (OA) as an inhibitor. As illustrated in Scheme 1, TiO_2_ was synthesized by calcination of the precursor obtained by OA-controlled hydrolysis of TBOT. In order to explore the effect of OA on the phase transformation of TiO_2_, the crystalline forms of TiO_2_ with different molar ratios of OA to TBOT at different calcination temperatures (CT) were investigated. When the ratios were 2:10, 5:10, 15:10 and 25:10, the precursors were calcined at 450, 550, 650 and 750 °C. To evaluate the photocatalytic performances of the synthesized TiO_2_ crystals, photocatalytic degradation experiments of MO and TC (as water pollutants) were carried out under light irradiation. Furthermore, the specific surface area, the recombination of photo-induced charge carriers and energy bands of the TiO_2_ crystals were analyzed in order to study the photocatalytic enhancement mechanism produced by the addition of OA.

## 2. Materials and Methods

### 2.1. Chemicals

Tetrabutyl titanate (TBOT, 98%, CAS: 5593-70-4), ethanol (99.5%, CAS: 64-17-5), oxalic acid (OA, 99%, CAS: 144-62-7), methyl orange (MO, 96%, CAS: 547-58-0) and tetracycline (TC, 96%, CAS: 64-75-5) were supplied by Aladdin Chemicals Co., Ltd. (Shanghai, China). Ammonium oxalate (AO, 99.8%, CAS:1113-38-8), 1,4-benzoquinone (BQ, 97%, CAS:106-51-4) and tert-butanol (TBA, 99.8%, CAS: 75-65-0) were purchased from Chengdu Chron Chemicals Co., Ltd. (Chengdu, China). All chemicals were analytical grade and used without further purification. The experimental water was deionized.

### 2.2. Preparation of Nano-TiO_2_ Samples

The synthesis process of nano-TiO_2_ crystals can be divided into 2 steps. The first step is to obtain the precursor solution by mixing solution A and B. Solution A was the TBOT ethanol solution with a concentration of 0.5 M. Solution B was a 60 mL mixture of 0.5 M OA aqueous solution and H_2_O. The increasing molar ratios of OA to TBOT, from 0:10 to 25:10, were accomplished according to the proportions of solutions A and B in Table S1. Firstly, to retard the hydrolysis rate and obtain a uniform and controllable precursor, solution B was added to solution A under the stirring in an ice-water bath (3–5 °C) for 3 h. Then, the mixture was placed in a 90 °C water bath for 8 h. The precursor solution was aged for about 20 h at room temperature (RT) to form a layered solution. After removing the upper layer, the milky white suspension in the lower layer was dried in an oven at 80 °C for about 6 h to obtain a white precursor solid. The dried precursor solid was ground into powder. Then, the powder was calcined in air at 450, 550, 650 and 750 °C with a heating rate of 5 °C/min and soaking time of 2 h. Finally, the samples were cooled to RT naturally.

### 2.3. Photocatalytic Activity Measurements

The photocatalytic performance experiments of TiO_2_ were carried out by using a xenon lamp (250 W, with irradiation intensity of 35 mW/cm^2^) as the light source to simulate sunlight. The UV–Vis absorption spectra were obtained using a UV-3600 UV–Vis spectrophotometer(UV, Shimadzu Group Company, Kyoto, Japan). MO and TC solutions were prepared with an initial concentration of 20 mg/L: 0.1 and 0.05 g of TiO_2_ samples were added to 20 mL MO and TC solutions, respectively. Before turning on the light source, we magnetically stirred the suspension for 30 min to establish adsorption–desorption equilibrium in the dark. After that, 1 mL of the solution was extracted per 15 min. The absorbances of MO at λ = 470 nm and TC at λ = 220 nm were tested using the supernatant after centrifugation. The degradation ratios (*D*) were calculated based on Formula (1).

(1) $$D=\frac{\left(A_{0}-A_{t}\right)}{A_{0}}\times 100\%=\frac{\left(C_{0}-C_{t}\right)}{C_{0}}\times 100\%$$

In the formula, *A*_0_ and *C*_0_ represent the initial absorbance and concentration of MO or TC, respectively. *A*_t_ and *C*_t_ represent the absorbance and concentration of MO and TC at time t, respectively.

### 2.4. Characterization of Nano-TiO_2_ Samples

The X-ray diffraction pattern (XRD) was obtained using an X-ray diffraction spectrometer (XRD, Dandong Haoyuan Instrument Co., Ltd., Dandong, China) with a radiation source of Cu (Kɑ), a tube voltage of 20 KV and a tube current of 30 mA. A scanning electron microscope (SEM, FEI Corporation, Hillsboro, FL, USA) was used for micromorphological analysis. Interplanar spacing analysis was performed by transmission electron microscopy (TEM, Japan Electronics Co., Ltd., Tokyo, Japan). Surface area measurements were performed on nitrogen adsorption-desorption data using Brunauer–Emmett–Teller (BET, Mike Instrument Company, Atlanta, GA, USA). X-ray photoelectron spectra were obtained with an X-ray photoelectron spectrometer (XPS, Kratos Ltd., Manchester, UK) for analyzing the elemental compositions and chemical states of the surfaces of the samples. In addition, the XPS spectrum of TiO_2_ was corrected on the basis of the C1s peak (284.8 eV). Ultraviolet-visible diffuse reflectance spectra (DRS, Shimadzu Group Company, Kyoto, Japan) were collected using a spectrophotometer. Photoluminescence spectra (PL, Hitachi, Ltd., Hitachi, Japan) were recorded on an F-4600 fluorescence spectrometer.

## 3. Results and Discussion

### 3.1. The Influence of OA on the Morphology of the Precursors

When the molar ratios of OA to TBOT were 0:10, 5:10, 15:10 and 25:10, the obtained precursor powders were named R0, R5, R15 and R25. The crystal structures of the precursor powders (Rx) were studied. From the XRD spectra of Rx in Figure S1, no obvious characteristic peaks of crystals were observed. Only a broad peak in the range from 15–20° was attributed to the contamination of the amorphous C on the measurement glass slide. Thus, it can be judged that the precursors were all non-crystalline. The HR-TEM images with no obvious crystal lattice and the corresponding Fast Fourier transform (FFT) image with a halo in Figure S2 confirmed the amorphous state of the precursors.

**Figure 1:**
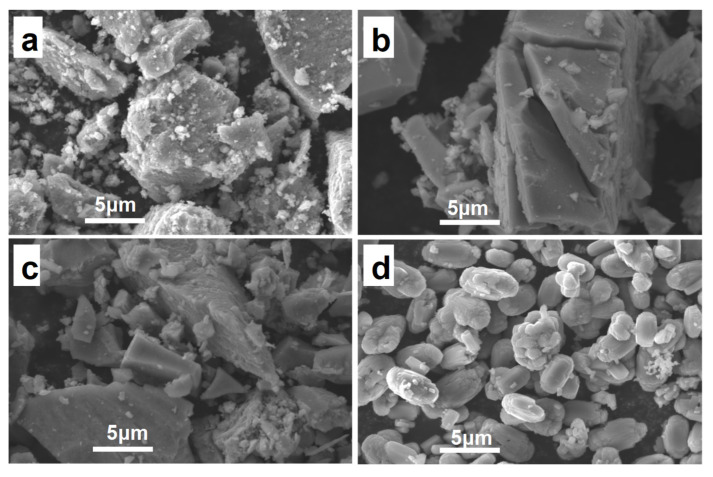
SEM images of the precursors: (**a**) R0, (**b**) R5, (**c**) R15 and (**d**) R25.

The SEM images of the amorphous precursors are shown in Figure 1. In Figure 1a, the irregular polyhedrons, flakes and numerous small particles can be observed in sample R0. The samples R5 and R15 had an irregular shape but relatively smooth-surfaced particles (Figure 1b,c). When the molar ratio reached 25:10, well-dispersed ellipsoidal grain-shaped particles were obtained; see Figure 1d. It can be seen that the addition of OA during the hydrolysis of TBOT can control the growth mode and improve the mono-dispersion of the precursors.

### 3.2. Controllable Phase Transformation of Nano-TiO_2_ Crystals Regulated by OA

**Figure 2:**
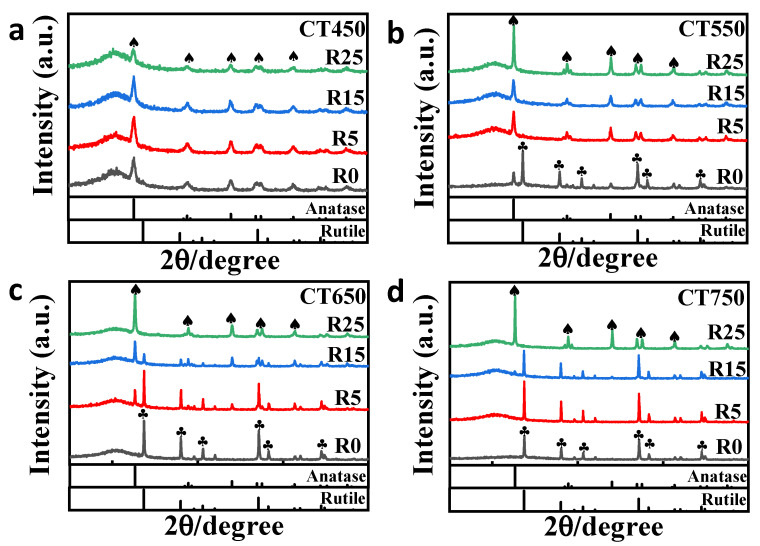
XRD patterns of TiO_2_ obtained from precursors R0, R5, R15 and R25 after calcination at (**a**) 450 °C, (**b**) 550 °C, (**c**) 650 °C and (**d**) 750 °C.

Next, the amorphous precursor powders Rx were calcined at 450, 550, 650 and 750 °C. The obtained samples were named CTy-Rx. The crystal structures of samples CTy-Rx were investigated by XRD (Figure 2). After calcination at 450 °C (Figure 2a), the samples CT450-R0, R5, R15 and R25 exhibited XRD peaks at 2θ = 25, 38, 48 and 55°, which we ascribed to typical anatase TiO_2_ crystal planes of (101), (103), (200) and (211) according to PDF#89-4921 (marked as ♠). Given the relatively weak intensities of the peaks, the samples were considered to be not well-crystallized, especially CT450-R25.

When the CT was 550 °C, the diffraction peaks in Figure 2b of all samples became sharper, indicating enhanced crystallinity. All samples also had the same characteristic diffraction peaks of anatase as those at 450 °C. Note for the sample CT550-R0, additional peaks at 2θ = 27, 36, 41, 44, 56 and 62° emerged, indexing to (110), (103), (200), (111), (002) and (301) planes of rutile TiO_2_ (PDF#99-0090, marked with ♣). Thus, as the CT was elevated, the obvious phase transformation from anatase to rutile first appeared in R0, where no OA was used during the hydrolysis of TBOT. As for R5, R15 and R25 with higher [OA], the calcined TiO_2_ still preserved the anatase phase.

From the results of the XRD spectra in Figure 2c, the sample of CT650-R0 was completely converted to rutile from anatase. As the molar ratio of OA to TBOT increased to five, the anatase phase was maintained in the mixed phase, although most crystals were rutile. As for the sample CT650-R15, the anatase peaks dominated the XRD peaks, leaving relatively weak rutile peaks. CT650-R25 was still anatase.

When the CT reached 750 °C, the characteristic peaks (Figure 2d) of rutile became sharper and those of anatase disappeared for the CT750-R0, CT750-R5 and CT750-R15 samples, indicating the phase of obtained TiO_2_ after calcination completely transformed from anatase to rutile. However, the R25 sample not only continued to maintain the crystal form of anatase, but had improved crystallinity. For the same precursor calcined at different temperatures, the evolution of the XRD spectra is also exhibited in Figure S3. It is shown obviously that precursors R0, R5 and R15 all experienced phase transformation from anatase to rutile at elevated CTs, from 450 to 750 °C, which agrees with the previous studies [36,37,38].

When the CT was 650 °C, the controllability of the phase transformation of TiO_2_ crystals by OA was fully reflected. Thus, the properties of these samples were focused on. The weight fractions of rutile and anatase phase (W_R/A_) for the mixed-phase TiO_2_ were calculated according to Formulas (2) and (3), where I_R_ and I_A_ represent the intensities of the characteristic diffraction peaks corresponding to the rutile (110) and anatase (101) crystal planes, respectively [36].

W_R_ = I_R_/(0.884I_A_ + I_R_) (2)

W_A_ = 1 − W_R_ (3)

As results calculated in Table 1, the W_A_ of CT650-R5 and CT650-R15 were 21% and 46%. As for CT650-R25, the W_A_ increased to 100%. The results of the XRD test also showed that the grain sizes of anatase and rutile crystals were nanoscale: 47.0 and 21.4 nm, respectively. Thus, the W_A_ in the obtained nano-TiO_2_ can be adjusted from 0 to 100% by simply adding OA and without changing the CT.

**Figure 3:**
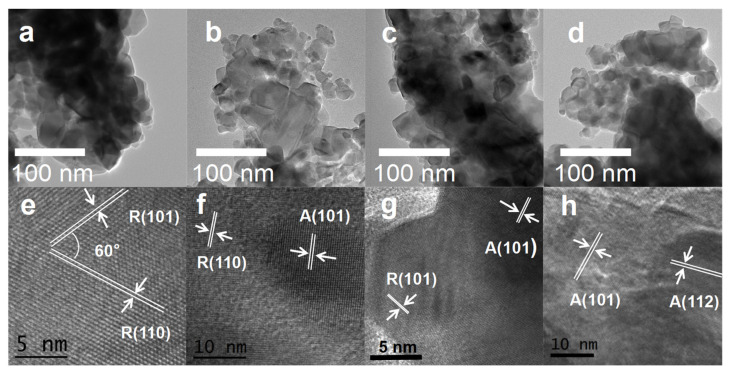
TEM and HR-TEM images of nano-TiO_2_ from (**a**,**e**) CT650-R0, (**b**,**f**) CT650-R5, (**c**,**g**) CT650-R15 and (**d**,**h**) CT650-R25.

These nano-TiO_2_ were also investigated by TEM and HR-TEM; see Figure 3. It is shown that the samples all consisted of a large number of nanocrystals. The particle sizes decreased when the concentration of OA increased. From the HR-TEM image of CT650-R0 in Figure 3e, the uniform interplanar spacings of 3.25 and 2.46 Å were ascribed to the (110) and (101) crystal planes of rutile, respectively. From Figure 3f, a small amount of anatase (101) crystals was found in the middle of the rutile (110) crystal. Figure 3g shows that both anatase and rutile crystal planes were recognized in sample CT650-R15. In Figure 3h, the nano-TiO_2_ particles of T650-R25 have uniform anatase crystal planes on the surfaces of the multiple-layer structures.

In all, with increased [OA], the temperature of phase transformation from anatase to rutile TiO_2_ can be elevated. Thus, the OA was found to enhance the thermal stability of the anatase phase of TiO_2_ by ensuring smaller crystal sizes from the analysis of nanostructures and XRD data.

### 3.3. Analysis of Photocatalytic Performance of TiO_2_ Regulated by OA

**Figure 4:**
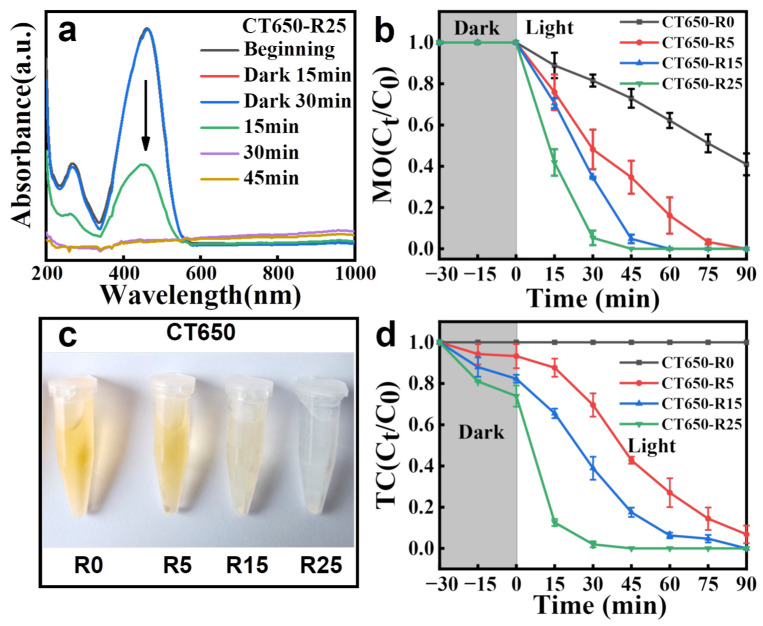
The photocatalytic activity of TiO_2_ regulated by OA. (**a**) The absorption spectra of MO degraded by CT650-R25. (**b**) The varied degradation rate of MO under light irradiation, (**c**) graph of the supernatants reacted for 30 min and (**d**) the evolution of the degradation rate of TC by using catalysts CT650-R25, CT650-R15, CT650-R5 and CT650-R0.

In order to evaluate the photocatalytic performance of nano-TiO_2_ regulated by OA, the samples calcined at 650 °C were selected for the degradation of MO and CT. After dark reaction for 30 min, the absorbance spectra (Figure S4) of MO in the solution were recorded every 15 min. For all of the nano-TiO_2_ samples, no obvious degradation or adsorption under dark reaction conditions was observed. As for the degradation processes under light irradiation, the absorbance of MO decreased to various extents. Figure 4a shows the greatest decrease in MO absorption when using the CT650-R25 sample as photocatalyst. The variations in the degradation ratio (D) vs. reaction time by using CT650-R0, CT650-R5, CT650-R15 and CT650-R25 as catalysts are shown in Figure 4b. When the degradation time was 30 min, the D were 22.0%, 50.8%, 65.7% and 98.7%, respectively. The corresponding photograph of supernatants is demonstrated in Figure 4c. The significantly faded orange represents the enhanced degradation abilities of TiO_2_ regulated by increasing OA. The CT650-R5, CT650-R15 and CT650-R25 samples completed the degradation in about 90, 60 and 45 min. The degradation reaction rate constants of MO degraded by CT650-R0, R5, R15 and R25 were calculated to be 0.010, 0.042, 0.066 and 0.098 min^−^^1^ from the linear relationships of −ln(C_t_/C_0_) vs. time in Figure S5. The degradation reaction rate constants of CT650-R25 were about 10 times that of CT650-R0. Thus, the photocatalytic performances of samples enhanced obviously as the [OA] in precursors and the WA of corresponding nano-TiO_2_ crystals increased.

The degradation experiments of TC were also performed. The evolution of the absorbance spectra of TC in the supernatants is shown in Figure S6. Figure 4d shows the corresponding degradation rates of TC as the catalytical reaction proceeded. It was found that the concentration of TC decreased gradually. When illuminated for 30 min, the D of the samples became 0, 9.6%, 35.1% and 59.7%, respectively. When the illumination time reached 90 min, the D of the samples were 0, 41.3%, 62.1% and 77.3%, respectively. Calculated in the same way as for Figure S7, the degradation reaction rate constants of TC degradation by CT650-R0, R5, R15 and R25 were 0, 0.031, 0.044 and 0.131 min^−^^1^, respectively. It was confirmed again that the higher the OA content in the precursor, the higher the photocatalytic activity of the calcined nano-TiO_2_ samples. The degradation effect of sample T650-25 was still the best.

### 3.4. The Mechanism of the Enhanced Catalytic Performance of TiO_2_

**Figure 5:**
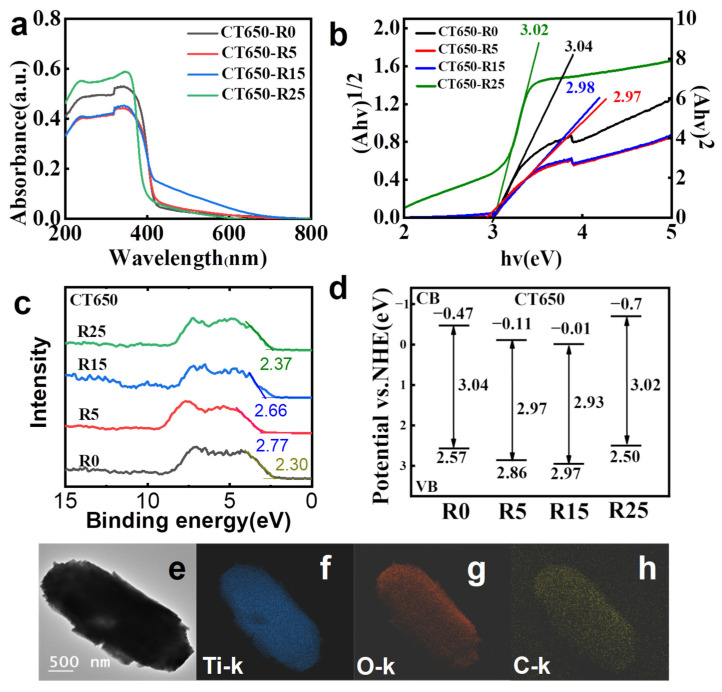
The mechanism of enhanced photocatalytic performance. (**a**) UV–Vis DRS spectra, (**b**) the h*v* vs (*α*h*v*)^1/2^ and (*α*h*v*)^2^ curves with marked band gaps, (**c**) VB-XPS spectra and (**d**) band structure diagrams of the CT650-R0, CT650-R5, CT650-R15 and CT650-R25 samples. (**e**) TEM image elemental mapping of (**f**) Ti, (**g**) O and (**h**) C of a discrete grain of CTR-25.

In order to reveal the mechanism of the enhanced catalytic performance of the nano-TiO_2_ regulated by increasing [OA], the E_g_ of samples CT650-R0, CT650-R5, CT650-R15 and CT650-R25 were analyzed firstly based on the DRS in Figure 5a. All samples demonstrated relatively high absorption for UV light. Compared with other samples, CT650-R25 had a higher absorption band in the UV region. The E_g_ of the photocatalysts can be calculated by Formula (4) [10]:

*α*h*v* = A(h*v* − E_g_)^n^ (4)

where *α*, h, *v* and A are the absorption coefficient, Planck’s constant, the frequency of light and the proportionality constant. The type of transformation determines the value of the exponent n, where values of 2 and 1/2 correspond to direct and indirect bandgap transformations, respectively [38]. Rutile and anatase/rutile mixed crystal were judged as having direct gap conversion, whereas pure anatase showed indirect band gap conversion [39,40,41]. A tangent line intersects the *x*-axis along the climbing part of the curve in Figure 5b. Thus, the E_g_ values of CT650-R0, CT650-R5, CT650-R15 and CT650-R25 were judged to be 3.01, 2.97, 2.93 and 3.00 eV, respectively. From the results, the E_g_ of the obtained TiO_2_ samples with the anatase phase almost decreased, relative to theoretical and experimental E_g_ values, by 3.2–3.59 eV [30]. Thus, our obtained mixed-phase and anatase TiO_2_ can allow a wider range of wavelengths for the activation process. Additionally, the excitation process of electrons from the valence band (VB) to the conduction band (CB) can be facilitated, thereby increasing the photocatalytic activity [42]. This improvement can be explained by the C doping in the anatase [43]. The elemental distribution of the “grains” in CT650-R25 sample is analyzed in Figure 5e–h. Apart from the Ti (blue) and O (red) elements covering the grain, the concentrated and obvious distribution of C (yellow) in the whole area of the particle was also detected. Carbon is an attractive dopant for TiO_2_, as it has been reported to reduce the band gap and improve photocatalytic performance in anatase [44]. Thus, OA molecules were thought to be doped homogeneously during the hydrolysis process of TBOT and control the growth mode of the precursors. Furthermore, the presence of OA in precursors can provide C and O doping sources in the calcination process of TiO_2_, regulating the crystallization and phase transition process.

The XPS spectra of the CT450-R25, CT550-R25, CT650-R25 and CT650-R25 samples are exhibited in Figure S8. The high-resolution (HR) spectra of Ti 2p are shown in Figure S9. The binding energies of Ti 2p3/2 (458.5 eV) and Ti 2p1/2 (464.2 eV) show that the Ti element had +4 valence [44]. The O 1s HR spectrum in Figure S10 shows the characteristic peaks at 529.4 and 530.0 eV, representing lattice oxygen (O^2−^) and a surface hydroxyl (OH^−^) [45], respectively. The HR spectra of C 1s are shown in Figure S11. The binding energies at 284.8, 285.8 and 288.5 eV were ascribed to C–C (C-H), C–O and C=O [46]. Comparing the HR spectra of these samples, we can see that the elevated temperatures had little effect on the element valance of Ti, O and C. The XPS valence band spectra (VB-XPS) for the samples are recorded in Figure 5c. A tangent line was drawn along the ascending part of the curve, and the abscissa of the intersection with the horizontal line is marked as the estimated valence band (E_VL_) of the sample. The E_VL_ of the samples were measured to be 2.37, 2.66, 2.77 and 2.30 eV, respectively. The real VB value needs to be obtained according to Formula (5) [47]:

E_NHE_ = Φ + E_VL_ − 4.44 (5)

In the formula, E_NHE_ and Φ are the normal hydrogen electrode potential and the work function of the instrument (4.6 eV), respectively. The final VB values were calculated to be 2.57, 2.66, 2.77 and 2.50 eV, respectively. Thus, the CB of these samples were found to be −0.47, −0.11, −0.01 and −0.7 eV, by subtracting the E_g_. Figure 5d is the schematic diagram of the VB and CB of each sample.

**Figure 6:**
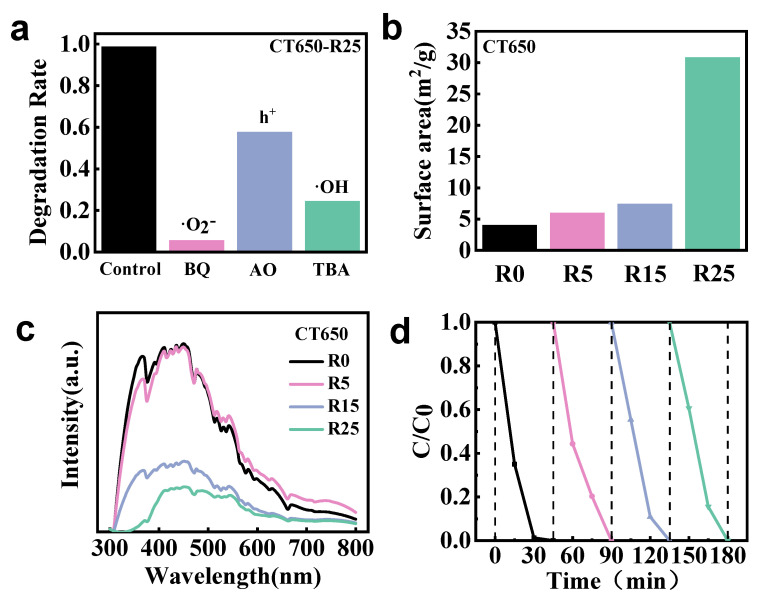
(**a**) The degradation rate of CT650-R25 in the presence of different scavengers. (**b**) The specific surface area and (**c**) PL spectra of CT650-R0, R5, R15 and R25. (**d**) Photocatalytic performance of recycled CT650-R25 catalyst.

The active species of CT650-R25 and CT650-R0 during the photodegradation process were studied. Ammonium oxalate (AO), 1,4-benzoquinone (BQ) and tert-butanol (TBA) [1] were added to capture superoxide radical species ·O_2_^−^, h^+^ and hydroxyl radicals (·OH), respectively. The different degradation results of CT650-R25 are exhibited in Figure 6a. When AO, BQ and TBA were added, the D of MO decreased from 98.7% to 94.4%, 42.3% and 75.6%. The photogenerated electrons in CB can populate unoccupied orbitals of oxygen molecules, yielding a O_2_^−^, then react with H_2_O to form ·OH. The photo-induced holes accumulated in the valence band can also react with H_2_O to form ·OH [37]. Finally, the MO and TC can be degraded by ·OH. Thus, the oxidizing ability of photogenerated electron–hole of CT650-R25 is the strongest. The obvious electron spin resonance (ESR) signals of superoxide (·O_2_^−^) and hydroxyl (·OH) radicals trapped by DMPO in CT650-R25 dispersion under the Xe lamp are shown in Figure S12. Thus, the O_2_^−^ and ·OH radicals were considered as the main active species of CT650-R25. By comparison to CT650-R0 in Figure S13, the degradation rate decreased slightly when the radical scavengers were added.

For catalysts, a larger specific surface area means more abundant catalytic binding sites, which are more conducive to the catalytic reaction. OA is a kind of polyoxyacid. In addition to regulating the crystal form, the effect of OA on the surface area cannot be ignored. The specific surface area data for each sample are shown in Figure 6b. The order of the specific surface area of TiO_2_ was CT650-R25 > CT650-R15 > CT650-R5 > CT650-R0. As the [OA] increased, the specific surface area of nano-TiO_2_ increased. The order of the specific surface area data is consistent with the order of the photocatalytic activity in Figure 4, which further confirms that OA addition improves the performance of nano-TiO_2_.

Since the energy after the recombination of photo-excited e^−^-h^+^ pairs will be released in the form of photoluminescence (PL), the PL spectra of the TiO_2_ samples were recorded in Figure 6c. It can be seen intuitively that the peak intensity of CT650-R25 is the lowest, which means that the recombination rate of e^−^-h^+^ pairs of this sample was the lowest. The peak fluctuation of CT650-R15 is significantly lower than that of CT650-R5 and CT650-R0. The peak area of CT650-R5 is slightly lower than that of CT650-R0. The perspective of fluorescence quantum yield is basically consistent with the reverse order of photocatalytic activity of the samples in Figure 4. The results show that the increased [OA] resulted in superior photocatalytic performance. This can be attributed to the reduction in the recombination process of the photo-induced charge carriers [39,48]. For further industrial applications, the stability of CT650-R25 was studied by performing four cycles of experiments. As shown in Figure 6d, although there might be some performance degradation, the MO was all removed within 45 min, suggesting considerable catalytic stability.

Therefore, the addition of OA can effectively control the crystalline forms, structure and photocatalytic performance of TiO_2_. The further study showed that OA could enhance the photocatalytic performance of TiO_2_ mainly in three aspects. First, the addition of OA can effectively reduce the E_g_ of TiO_2_ by C doping in the crystals. Second, the addition of OA can increase the specific surface area of TiO_2_, indicating more abundant the catalytic binding sites. Third, the more OA added, the less the photo-induced charge carriers are recombined, thereby improving the catalytic utilization of carriers.

## 4. Conclusions

In conclusion, controllable adjustment of the dispersion and transformation temperature of crystalline forms and photocatalytic performances of the nano-TiO_2_ samples was achieved by using OA. The study shows that the addition of OA can prevent the crystalline form’s transformation from anatase to rutile effectively, which is important for the applications of anatase at high temperatures. The transformation temperature increased gradually when increasing the ratio of [OA] in hydrolysis process of TBOT. At the same CT (650 °C), when the molar ratio of OA to TBOT increased from 2:10, to 5:10, to 15:10 to 25:10, the W_A_ was tuned from 0% to 100%, and the photocatalytic activity could be significantly enhanced. The mechanism study showed that an increased [OA] can significantly improve the dispersibility of the catalyst, increase the specific surface area, reduce the recombination rates of photo-induced charge carriers and decrease the E_g_ by doping C. Thus, the purpose of improving the photocatalytic performance was achieved. This work provides a mild and simple synthetic method for controlling the crystalline forms and photocatalytic performance of nano-TiO_2_ effectively.

## Figures and Tables

**Table 1:** The weight fractions and crystallite sizes of rutile and anatase phases of CT650-R0, R5, R15 and R25.

Samples	Anatase		Rutile	
	WA(%)	Crystallite Size(nm)	WR(%)	Crystallite Size(nm)
CT650-R0	-	-	100	47.0
CT650-R5	21	37.4	79	33.2
CT650-R15	46	29.9	54	29.7
CT650-R25	100	21.4	-	-

## Data Availability

Data are contained within the article.

## Supplementary Materials

The following supporting information can be downloaded at: https://www.mdpi.com/article/10.3390/nano12173019/s1, Table S1: the dosages of solution A and B with different molar ratios of OA to TBOT; Figure S1: XRD patterns of precursors R0, R5, R15 and R25; Figure S2: the TEM, HR-TEM and corresponding Fast Fourier transform (FFT) images of the precursor R0 and R25; Figure S3: XRD patterns of TiO_2_ obtained from precursors R0, R5, R15 and R25 after calcination at 450 °C, 550 °C, 650 °C and 750 °C; Figure S4: the absorbance of MO degraded by the CT650-R0, CT650-R5 and CT650-R15 samples; Figure S5: the −ln(C_t_/C_0_) of MO versus time degraded by the CT650-R0, CT650-R5, CT650-R15 and CT650-R25 samples; Figure S6: the absorbance of TC degraded by the CT650-R5, CT650-R15 and CT650-R25 samples; Figure S7: the −ln(C_t_/C_0_) of TC versus time degraded by the CT650-R0, CT650-R5, CT650-R15 and CT650-R25 samples; Figure S8: XPS total spectra of CT450-R25, CT550-R25, CT650-R25 and CT750-R25; Figure S9: HR XPS spectra of Ti 2p of CT450-R25, CT550-R25, CT650-R25 and CT750-R25; Figure S10: HR XPS spectra of O 1s of CT450-R25, CT550-R25, CT650-R25 and CT750-R25; Figure S11: HR XPS spectra of C 1s of CT450-R25, CT550-R25, CT650-R25 and CT750-R25; Figure S12: ESR spin signals of superoxide (·O_2_^−^) and hydroxyl (·OH) radicals trapped by DMPO in CT650-R25 dispersion under the Xe lamp; Figure S13: the degradation rate of CT650-R0 in the presence of different scavengers.
