# Supplementary material for: Controllable Phase Transformation and Enhanced Photocatalytic Performance of Nano-TiO2 by Using Oxalic Acid
Source: Nanomaterials (Basel). 2022 Aug 31;12(17):3019. doi: 10.3390/nano12173019 (PMC9458251; doi:10.3390/nano12173019)
Supplement: Supplementary file 1 [file nanomaterials-12-03019-s001.zip › nanomaterials-1859026-supplementary.pdf]

Controllable Phase Transformation and Enhanced Photocatalytic Performance of Nano-TiO_2 by Using Oxalic Acid

Jiaqi Chen [1], Jian Gao [1], Xiaoyang Liu [1], Pan Wang [1], Xue Yu [1], Feng Zhao [2], Yan Sun [1], Wei Feng [1,*] and Qingyuan Wang [1,*]

[1] School of Mechanical Engineering, Chengdu University, Chengdu 610000, China
[2] Institute for Advanced Materials Deformation and Damage from Multi-Scale, Chengdu University, Chengdu, 610106, China
* Correspondence: fengwei233@126.com (W.F.); wangqy@cdu.edu.cn (Q.W.)

Table S1. The dosages of solution A and B with different molar ratios of OA to TBOT.

Solution / Ratio	Solution A (mL)	Solution B (mL)	
		OA (mL)	H_2O (mL)
**0：10**	20	0	60
5：10	20	10	50
15：10	20	30	30
25：10	20	50	10

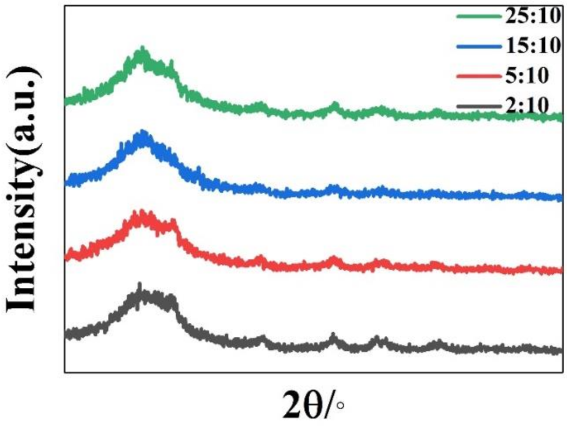

Figure S1. XRD patterns of precursors R0, R5, R15 and R25.

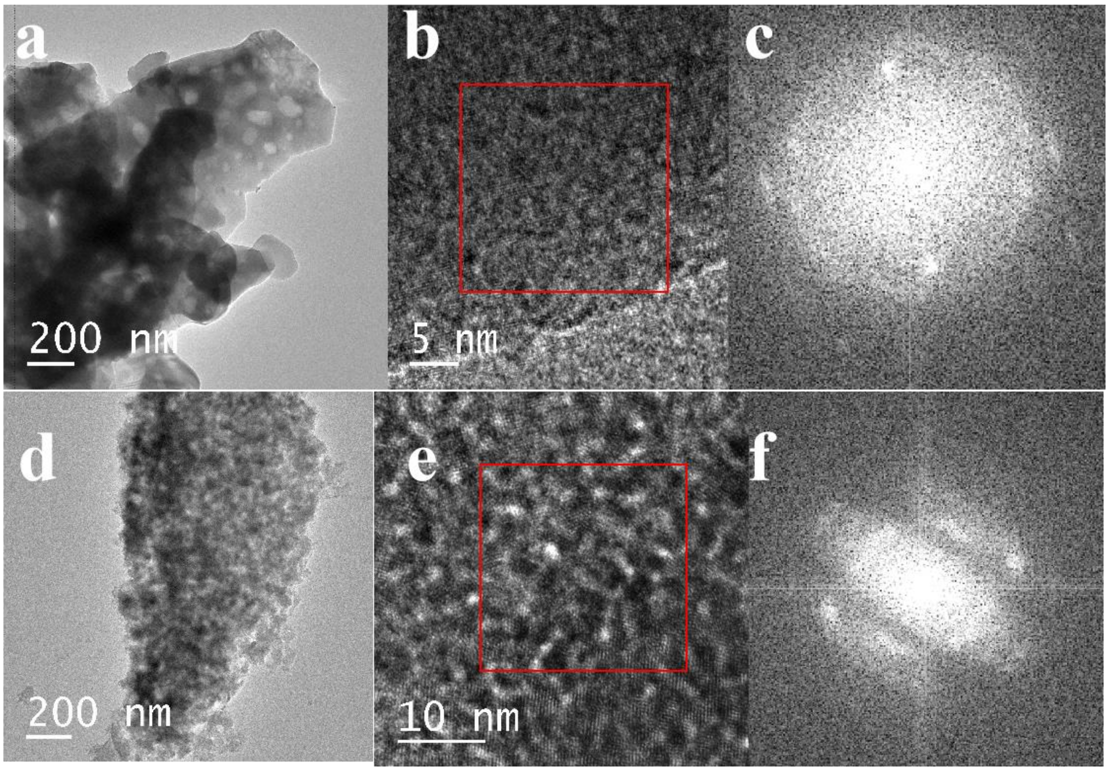

Figure S2. The (a, d)TEM, (b, e) HR-TEM and (c, f) corresponding Fast Fourier transform (FFT) images of the precursor R0 and R25.

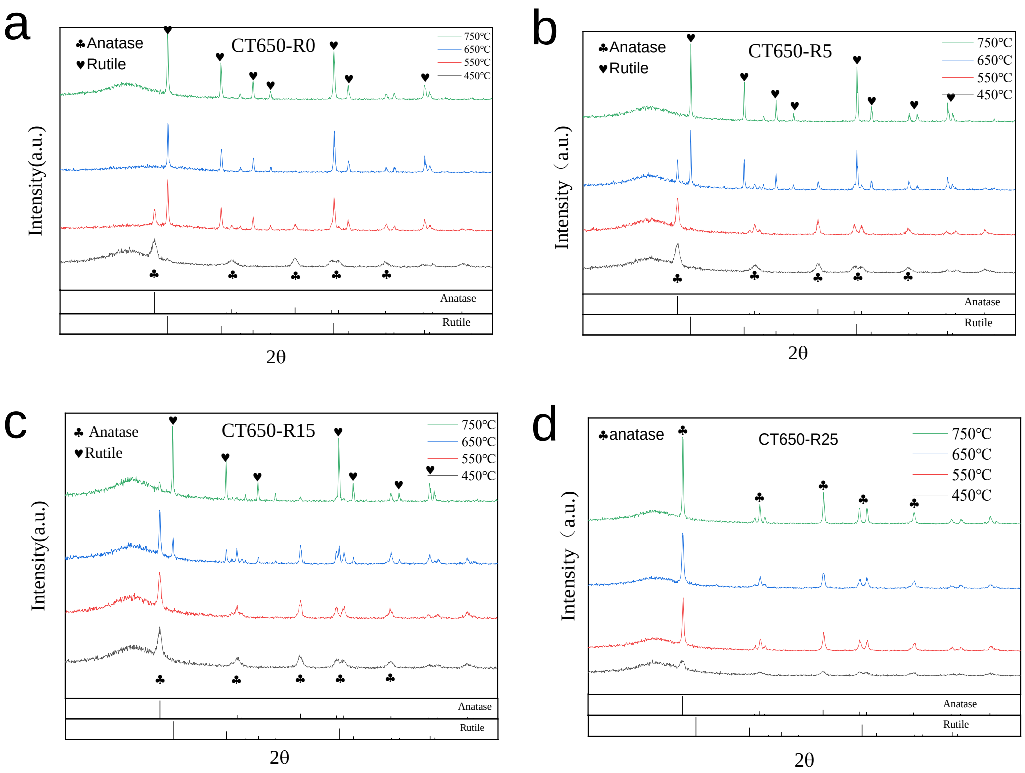

Figure S3. XRD patterns of TiO_2 obtained from precursors (a) R0, (b) R5, (c) R15 and (d) R25 after calcination at 450℃, 550℃, 650℃ and 750℃.

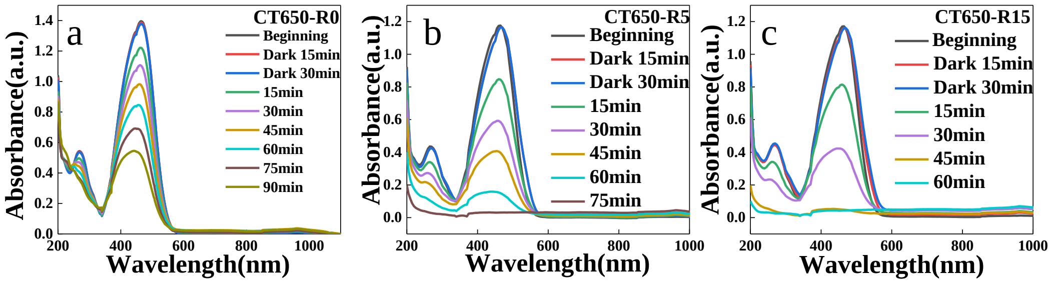

Figure S4. The absorbance of MO degraded by the CT650-R0, CT650-R5 and CT650-R15 samples.

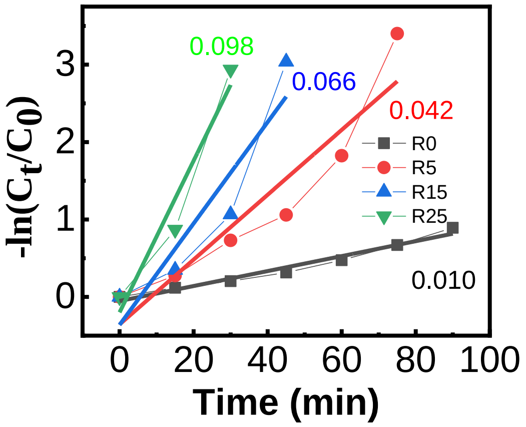

Figure S5. The -ln(C_t/C_0) of MO versus time degraded by the CT650-R0, CT650-R5, CT650-R15 and CT650-R25 samples.

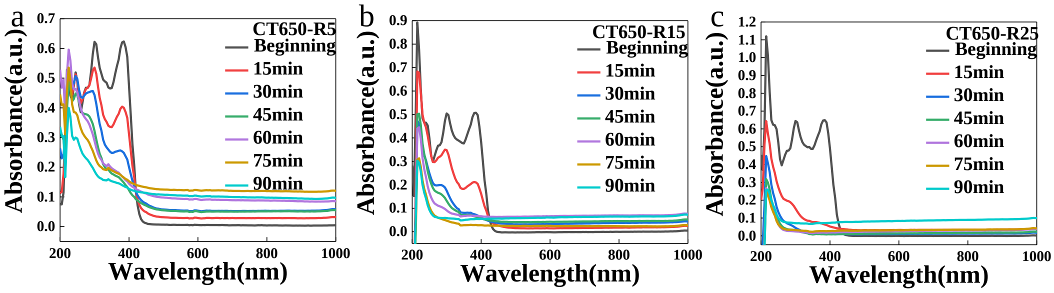

Figure S6. The absorbance of TC degraded by the CT650-R5, CT650-R15 and CT650-R25 samples.

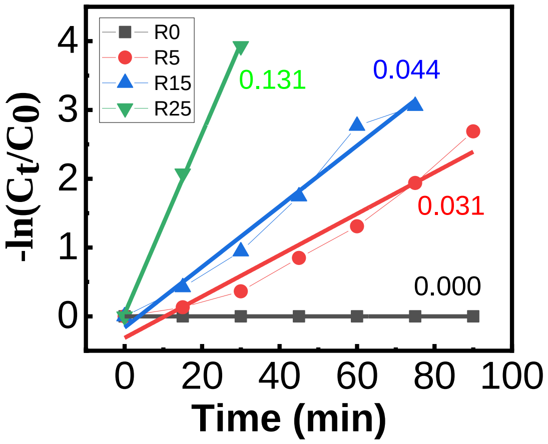

Figure S7. The -ln(C_t/C_0) of TC versus time degraded by the CT650-R0, CT650-R5, CT650-R15 and CT650-R25 samples.

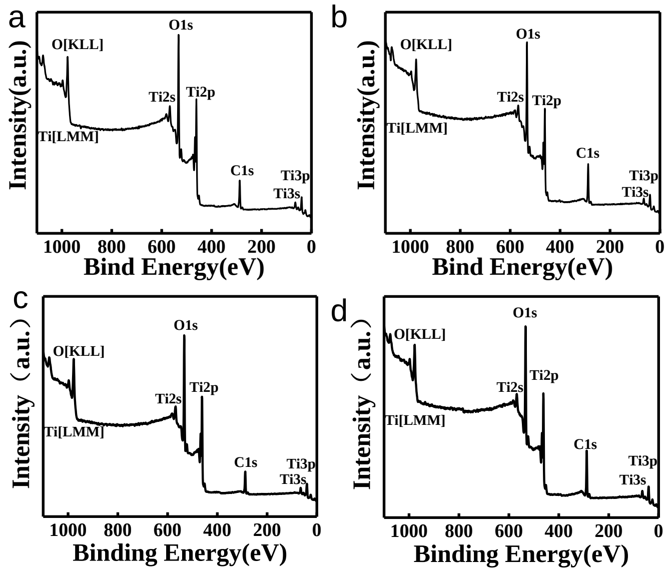

Figure S8. XPS total spectra of CT450-R25 (a), CT550-R25 (b), CT650-R25 (c) and CT750-R25 (d).

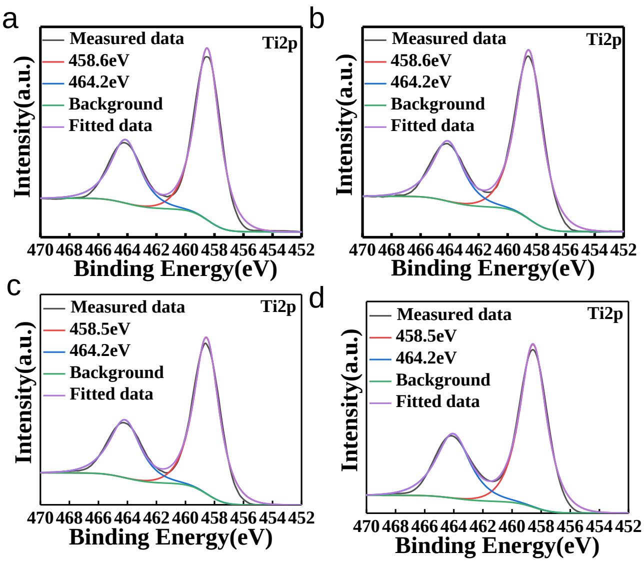

Figure S9. HR XPS spectra of Ti 2p of CT450-R25 (a), CT550-R25 (b), CT650-R25 (c) and CT750-R25 (d).

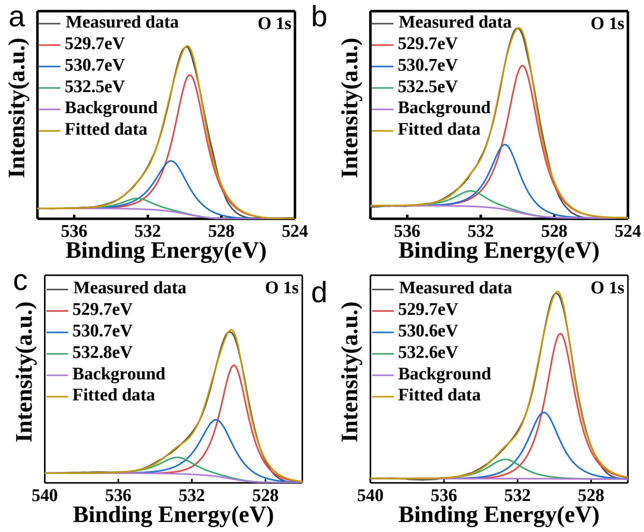

Figure S10. HR XPS spectra of O 1s of CT450-R25 (a), CT550-R25 (b), CT650-R25 (c) and CT750-R25 (d).

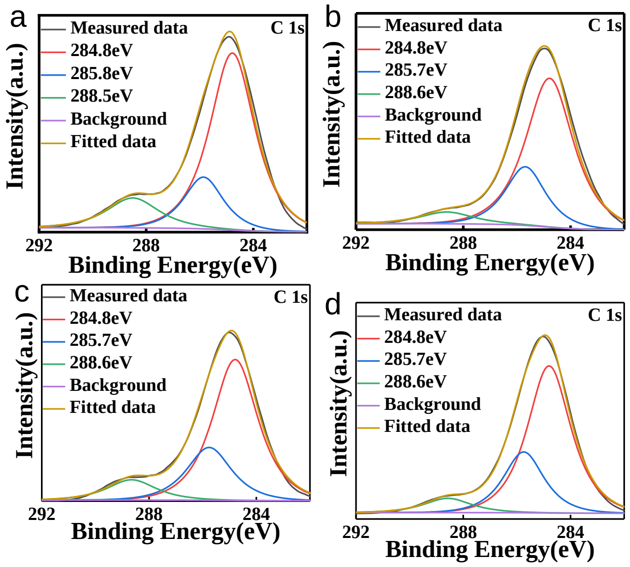

Figure S11. HR XPS spectra of C 1s of CT450-R25 (a), CT550-R25 (b), CT650-R25 (c) and CT750-R25 (d).

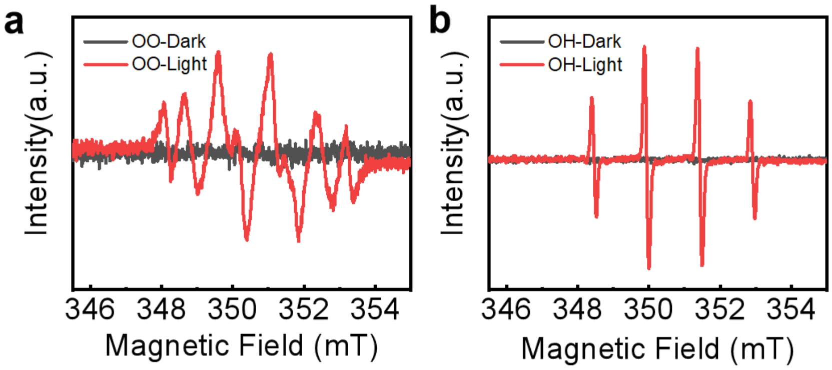

Figure S12. ESR spin signals of (a) superoxide (•O_2^-) and (b) hydroxyl (•OH) radicals trapped by DMPO in CT650-R25 dispersion under the Xe lamp.

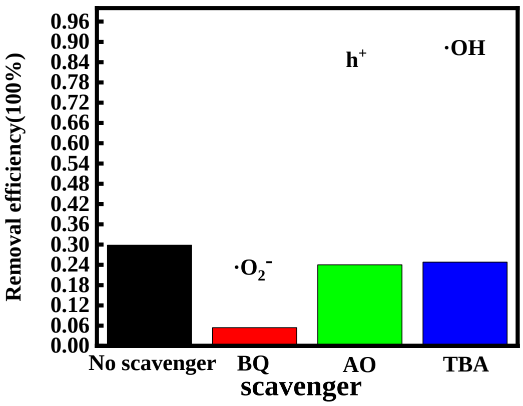

Figure S13. The degradation rate of CT650-R0 in the presence of different scavengers.
